# RoCS: Robotic Curriculum for young Surgeons

**DOI:** 10.1007/s11701-022-01444-3

**Published:** 2022-07-09

**Authors:** Jessica Stockheim, Aristotelis Perrakis, Bernhard A. Sabel, Robert Waschipky, Roland S. Croner

**Affiliations:** 1 Department of General, Visceral, Vascular and Transplant Surgery, University Hospital Magdeburg, Otto-Von-Guericke University Magdeburg, Leipziger Str. 44, 39120 Magdeburg, Germany; 2 Institute of Medical Psychology, Otto-Von-Guericke University Magdeburg, Magdeburg, Germany; 3 Department of Information Technology (IT) and Medical Engineering, Otto-Von-Guericke University Magdeburg, Magdeburg, Germany

**Keywords:** Robotic surgery, Training, NASA Task Load Index, Surgical curriculum, Residency

## Abstract

Robotic-assisted procedures gain increasing acceptance for daily surgical routine. However, structured training programs are designed for surgeons with high expertise. Hence, a comprehensive training curriculum was established to ensure a basic competence in robotic abdominal surgery for young surgeons during their residency. The aim of the current work is to propose a feasible and effective training concept. The development process of this training curriculum is based on a comprehensive literature review which led to the concept of “robotic curriculum for young surgeons” (RoCS). It was implemented in the daily routine of a German university hospital starting in 2020. The robotic assessment questionnaire (RAQ) was used for electronic data collection. After the initial phase adjustments, it led to an improvement of the initial version of the curriculum. RoCS is a multimodal training program containing basic training through assistance at the operation table during robotic-assisted operations and basic console training. Key elements are the robotic team time-out (rTTO), perioperative process standardization including feasible personnel scheduling and useful procedure clustering into organ systems, procedural steps and procedural step complexity. Evaluation of standardized communication, performance assessment, patient factors and individual overall workload using NASA Task Load Index is realizable. Flexibility and adaptability to internal organization processes of surgical departments are the main advantages of the concept. RoCS is a strong training tool to meet the specific needs of young surgeons and evaluate their learning success of robotic procedural training. Furthermore, comparison within the different robotic systems should be considered. Further studies are needed to validate a multicenter concept design.

## Background

Surgical procedures in visceral surgery have been revolutionized by robotic systems due to their high technical complexity and precision. The case numbers of robotic operations have been increased exponentially over the last two decades [1–3] and new robotic systems emerge to the market [3]. Currently the robotic systems are designed for surgeons with advanced and/or expert surgical skills, e.g., colorectal surgeons [4]. Nevertheless, the procedures will become more and more an integral part of daily routine [5]. Therefore, it is emendable to standardize training concepts [1] and refer them to the education of unexperienced surgeons during their residency (so called “young” surgeons) [6, 7]. Development of a feasible curricular structure which can be implemented into training programs of other facilities is a challenge referring to change management as an important and ongoing process [8, 9]. To develop a structured curriculum for robotic surgery, different requirements are essential to achieve basic robotic skills and console proficiency: a robotic training structure should separate basic and advanced robotic skill training with a modular approach to index surgical procedures [10]. Another core challenge is the conveyance of specific knowledge according to the robotic technique, intraoperative procedural steps and the ability of autonomous surgical performance including perioperative processes, such as patient positioning, port placement, docking, intraoperative assistance and console assistance and nontechnical skills [11]. Krause et al. presented a robotic training program which is divided in two phases and refers to a certain level of surgical residency [12]. Furthermore, institutional resources, individual qualities, and curricular elements should be addressed [13]. Dual robotic console models, a robotic-focused faculty, resident interest in robotic surgery, relevance and consideration of post-graduate year during the training process and simulator training are recommended [13]. Currently, there are only few standardized training programs for robotic-assisted surgical procedures. Moreover, training programs are often designed as workshops [14] or as fellowship programs [15] supported by institutions or societies [7, 16]. It was shown that residents’ satisfaction increases during participation in a voluntary program which is based on personal investment of time outside daily surgical routine [12]. Aradaib et al. report that experiences from the first 55 robotic surgeries showed a safe adoption of a structured training as documented by patient outcome and evaluation [16]. Admittedly, development of a training concept was a time-consuming process [16] when no compromises are made in terms of patient safety and surgical outcome [4, 16]. Team communication and training is essential especially with regard to technical aspects, in case of critical events considering the influence of individual workload [11, 17].

In summary, there are currently no reports about a comprehensive robotic surgery program for residents at an early stage of training which is applied to the broad spectrum of visceral surgery and which can be performed during daily routine and unites the above-mentioned training elements. Therefore, our goal was to create, test and establish a feasible robotic curriculum, the “Robotic Curriculum for young Surgeons (RoCS)”. It enables novice surgeons during their residency to gain basic robotic skills within regular working hours. The RoCS program includes a simultaneous back and forth evaluation between the involved disciplines and surgeons. In the interest of patient safety, the concept is based on precise surgical state-of-the-art performance of the procedures in an interdisciplinary robotic operating room (OR) setting including surgeons, nurses and anesthesia. Therefore, the aim of the present work is to propose a structure of a robotic training program for surgeons during their residency in visceral surgery.

## Materials and methods

The idea of a robotic curriculum for novice surgeons originated from considerable interest of residents in participation in robotic-assisted procedures and the respective training requirement defined by the leading robotic expert. Accordingly, a curricular team was assembled, consisting of one surgical expert, an experienced surgeon and a novice.

Because the process of change has to be managed strategically within a given healthcare setting [9], it is inevitable to consider change as natural, embrace it and sustain a culture of change [8, 9]. In respect to the principles of curriculum design [8], the following procedural steps describe the process of development and implementation of RoCS.

### Curricular objective and targets

The objective of the curriculum was to gain basic robotic competence in the ability to perform robotic bedside assistance and low and moderate complex procedural steps at the robotic console under supervision during robotic visceral operations.

The definition of outcome measures including surgical competency, nontechnical skills [11], progression, organizational and patient aspects is shown in Table 1. No compromise was made regarding the priority of patient safety and patient outcome, both of which were monitored and recorded as well as other patient data and perioperative parameters.

**Table 1:** Definition of outcome measures including competency, progression, organizational and patient aspects

Target	Explanation
Surgical competency	
Soft skills	Communication and feedback culture; rate of performed standardized communication
Practical skills	Successful performed simulator training due to predetermined exercises; intraoperative overall workload evaluation by NASA Task Load Index; ability of bedside assistance independently (OSCORE and residents’ feedback questionnaire); rate of independent performed intraoperative procedural steps at the robotic console and their complexity
Surgical progression	
Practical skills	Difference between planned/preoperative specified intraoperative procedural steps and actual performed intraoperative procedural steps; relation to surgical experience level
Organization	
Participant motivation	Repetitive data analysis for involved personnel to enhance learning progression including feedback and NASA Task Load Index results
Personnel scheduling	Successful implementation given by rate of scheduled and actual involved personnel to robotic procedures; achievement of targeted number of procedures
Patient safety	Assurance of state-of-the art surgical performance by standardization of robotic procedures; intraoperative overall workload evaluation by NASA Task Load Index; patient outcome including operation parameters, morbidity and mortality

### Needs assessment, analysis of local resources and literature review

Initially data on the current institutional structures and robotic training were collected and analyzed by the curricular team. This included technical, personnel and organizational resources, established structures and yearly robotic case load and daily challenges due to one da Vinci® operation robot. The current robotic teaching approach and educational system were assessed.

A comprehensive literature search was performed in PubMed from 2012 to 2022 using the following keywords and their combination: robotic visceral surgery, surgical training; curriculum, robotic surgery, general surgery. A critical evaluation of the results and retrieved papers was conducted by the curricular team. Inclusion criteria were: (1) robotic visceral surgery; (2) description of curricular structures in title or abstract. Publications were excluded in case of virtual settings/training and in case of specialties other than visceral or general surgery.

### Creation of the robotic training concept RoCS and evaluation methods

Based on the needs, resources and literature assessment the essential elements for the local curricular setting were identified: knowledge transfer theoretically and practically, communications skills, practical performance training, workload and teaching evaluation. The curricular concept combined these elements in a practical system of teaching and learning during daily routine. Evaluation methods were implemented accordingly. The Institute of Medical Psychology of Otto-von Guericke University Magdeburg reviewed evaluation methods.

The didactic key elements were defined referring to different teaching models of Zwisch-Model [18], briefing and debriefing method [19], the BID model [17], Dreyfus model of skill acquisition [20] and Halstead’s principle of surgical training and core competencies [21].

Communication skills were implemented in different ways. Scientific bases of feedback referred to the O-SCORE [22], the BE-SMART concept [23] and common feedback methods like ‘tip top’. Referring to the reverse feedback direction from resident to expert a modified questionnaire version was developed. It based on the O-SCORE with additional aspects due to specific training topics. Moreover, aspects of teaching and learning satisfaction were implemented. Referring to the TeamSTEPPS [24] model of surgical communication modification led to the *robotic team time-out (rTTO)*. It focused on amplifying the learning effect of each surgical OR team member.

The intraoperative workload was evaluated using the NASA Task Load Index (NASA TLX) [25–27], a simple tool with six questions (subscales rate from 0 to 20). The result value of the overall workload varies between 0 and 100. In addition, another question was complemented referring to the preoperative expectations of the difficulty level of the operation [26]. It was used as a monitor of learning progression [28], workplace environment and assurance of patient safety.

To determine the initial surgical experience and to evaluate its impact on the learning process, an experience score was developed, because surgical experience is a relevant factor due to robotic training and the level of experience needs to be recognized. Experience clusters were based on the number of performed operations. But in contrast to common literature, which defines experience mainly by counting years of practice [27], we classified expertise by a four-staged rating for each surgical approach as shown in Table 2: minimal/none, low, moderate, high. Referring to current robotic learning progress, the learning curve flattens after 50 performed procedures [29]. The differentiation by surgical approach was made additionally: open, laparoscopic, robotic. Consecutively, an overall experience score was determined by assigning ‘experience score count’ (ESC) from 0 to 3 to each experience level. Using this scoring system, the overall experience score varies between 0 and 9, and this results in three levels of surgical practical competency (*surgical experience level*): basic (0–3), advanced (4–5) and expert (6–9).

**Table 2:** Surgical experience levels defined by count of performed procedures differentiated by surgical method

Surgical approach	Experience level	Performed procedures (n)	Experience score count
Open	High	> 300	3
	Moderate	101–300	2
	Low	20–100	1
	Minimal/none	< 20	0
Laparoscopic	High	> 100	3
	Moderate	51–100	2
	Low	20–50	1
	Minimal/none	< 20	0
Robotic	High	> 50	3
	Moderate	31–50	2
	Low	15–30	1
	Minimal/none	< 15	0

To train robotic procedural techniques, the residents were instructed to use the da Vinci simulator^®^ weekly for individual duration. For teaching and standardization purposes, especially for assistance at the robotic console, the usually performed procedures were split into specific intraoperative procedural steps following the principle idea of sub-steps [30] in a modified way (simple, moderate complex and high complex steps).

### Participant and patient recruitment

The recruitment of participants was performed prospectively and included members of the surgical teams (OR nurses, surgeons) and patients undergoing robotic procedures as the targeted population. Exclusion criterion was the absence of informed consent.

### Electronic data collection in collaboration with referring department, future analysis

Electronic data collection was realized by Redcap^®^ with university license in cooperation with the local Department of Information Technology (IT) and Medical Engineering as the first project using RedCap^®^ at the University Hospital Magdeburg. After creation of the RoCS project, a web link was generated, which was used to collect data digitally. Statistical data exploration and analysis will be performed by RedCap^®^ and IBM^®^ SPSS Statistics 28.0.

### Initialization of RoCS with simultaneous evaluation and feedback loop

After receiving a positive vote of the local ethical commission (internal number 152/50), the preliminary version of RoCS was introduced to the interdisciplinary surgical team by oral presentation. The implementation of the project into daily routine was initiated in March 2020. It represents the first milestone with a consecutive timeline of twelve months. Personal and repetitive briefings in the OR during robotic procedures were also performed. Pocket card guidelines were printed and distributed as a reminder, which described the documentation steps for evaluation purposes and included key points of the concept as well as a web link for digital documentation. To maintain motivation, acceptance and curricular structures, repetitive reminders were sent by email to the participants every month. Furthermore, every participating surgeon attended and successfully completed a workshop of introduction to the robotic system instructed by Intuitive^®^. Online registration to the da Vinci Surgery Online Community^®^ was fundamental to obtain personal access to indivual simulator training sessions.

### Periodical concept and feedback analysis and consecutive adjustments

The time frame of the initial development phase was one year. After a scheduled period of six and twelve months, evaluations were made by internal assessment of personnel, organizational and conceptual aspects. Furthermore, during daily routine, personal feedback was given to the curricular team on an as-needed-basis. During the scheduled feedback sessions, information was retrieved from the involved surgeons by a three-item questionnaire (positive or problematic aspect of the concept, adjustment suggestion). In addition, the participating OR nurses and surgeons received a personal summary of overall workload and digital documented feedback referring to their operations. Apart from evaluation aspects, the scheduled meetings included communication training according to the curricular key elements supported by collaboration partners. Consequently, adjustments were implemented.

## Results

The initial assessment revealed various aspects which needed to be addressed for the development of a feasible training system at the surgical department at the University Hospital Magdeburg: OR capacities, one available operation robot (DaVinci X^®^; DaVinci Xi^®^ since 12/2020) with two consoles, one DaVinci^®^ training simulator, three surgical robotic experts, assessment of cost-effectiveness of robotic surgical procedures, and availability of digital data collection and evaluation.

The literature search of curricular programs for robotic visceral surgery provided a total of 74 results after removal of duplicates. Of these, 17 studies were identified which included description of curricular teaching structures for robotic visceral surgery [2, 4, 12, 14, 16, 31–43] (Table 3). The relevant studies were screened regarding authors, journal and year of publication, personnel participation, simulator training, bedside assistance, console training, referring operations or organ systems, curricular phases or time periods, connection to residents’ post-graduate year or any other connection to surgical experience and evaluation methods (Table 3).

**Table 3:** Literature overview and comparison of curricular programs for robotic visceral surgery

	Author	Journal, year	Personnel participation (General/visceral surgery)	Simulator training	Bedside assistance	Console training	Operations/Organ systems	Curricular phases/duration	Curricular connection to surgical experience level/Pgy^**1**^	Evaluation method
1	Grannan et al. [31]	Surg Laparosc Endosc Percutan Tech, 2021	Residents	Included	Included	Included	—	1	PGY 1	Post-curriculum survey (resident opinion)
2	Mustafa et al. [32]	J Surg Educ, 2019	Residents	—	—	Included	Hernia repairs	—	—	Retrospective case review
3	Moit et al. [33]	JSLS, 2019	Residents	Included	Included	Included	—	2	Phase 1: PGY 1–2 Phase 2: PGY 3–5	Global Evaluative Assessment of Robotic Skills for resident's operative performance
4	Krause, Bird [12]	J Robot Surg, 2019	Residents, voluntarily	Included	Included	Included	—	2	Phase 1: PGY 1–2 Phase 2: PGY 3 +	Survey (residents’ individual opinion) including 6 questions
5	Ramirez Barriga et al. [34]	J Surg Educ, 2022	Residents	Included	—	—	—	2 week period	PGY 3	Surveys, comparison to laparoscopic and open techniques, performed mentor sessions and exposure in the OR
6	Knab et al. [42, 43]	Ann Surg Oncol, 2018	Fellows	Included	—	Included	Pancreas resections	5 steps	Included	Depending on the step; including fellow’s education portfolio, pre-/ post-test design, OSATS score, video library, feedback, review of operative performance, etc
7	Gonzalez-Hernandez et al. [35]	J Robot Surg, 2018	Residents	Included	Included	Included	Hernia repairs	—	Included	Comparison of surgical experience and patient outcomes with/without resident participation
8	Winder et al. [36]	J Robot Surg, 2016	Residents	Included	Included	Included	CR^2^, biliary, foregut operations	1 year period	—	Education time and self-reported progress, rate of bedside assistance and operating at the console
9	Arain et al. [37]	Surg Endosc, 2012	Residents, interdisciplinary	Included	—	—	—	2 month period	Included	Task scoring for time and errors using modified FLS metric
10	Formisano et al. [38]	Minerva Chir, 2019	Attendings	—	Included	Included	Right hemi-colectomy	—	Included	Perioperative factors, pathological outcomes
11	Ebeling et al. [39]	Am J Surg, 2020	Residents and fellows	—	—	Included	Inguinal hernia repairs	—	—	Patient and operation characteristics, residents’ technical competency (GEARS), Zwisch scores, number of robotic console cases (residents as primary surgeon)
12	Juza et al. [40]	J Robot Surg, 2014	Residents and fellows	Included	Included	Included	Initially cholecystectomies	—	Novice robotic surgeons	Comparison to laparoscopic cholecystectomies
13	Aradaib et al. [16]	J Robot Surg, 2019	Colorectal surgeons	Included	—	Included	CR^2^	European Academy of Robotic Colorectal Surgery (EARCS) program	Robotic beginners	Multiple patient and perioperative characteristics, 30-day mortality, pathological outcome
14	Buchs et al. [14]	Surg Endosc, 2013	—Interdisciplinary	Included	Included	Included	—	2-day course	—	Retrospective review of the participants' surgical practice using online research and surveys
15	Rückbeil et al. [41]	Zentralbl Chir, 2022	Residents	—	—	Included	CR^2^	100 consecutive operations	—	Patient and perioperative characteristics
16	Collins et al. [2]	J Robot Surg, 2021	Residents, nurses, surgeons, medical students	—	Included	—	—	3 stages	—	Pre- and post-Robotic Assistant Surgical Training (RAST) survey (25 questions), between-stages repeated-measures survey (11 questions); including baseline demographics, confidence level, fundamental knowledge, participant feedback, etc
17	Thomas et al. [4]	J Robot Surg, 2021	Colorectal surgeons	Included	—	Included	CR^2^	1-year period	—	Comparison with prior surgical results of involved surgeons, patient and perioperative characteristics, pathological outcome

^1^*CR* colorectal surgery, ^2^*PGY* post-graduate year

Due to the robotic workplace, a measurable, standardized communication was inevitable to encourage a constructive atmosphere of teaching, learning and evaluation of the curriculum. Therefore, interdisciplinary cooperation with two collaborating departments was initiated to gain expertise, technical and curricular support and feedback. Furthermore, descriptive statistical analysis of involved personnel and simulator training was conducted by the curricular team during the initial twelve months: in this time frame, 107 robotic procedures were performed. Referring to RoCS, the assigned surgeons participated in 78.8% (82/107) as bedside assistance for liver, pancreas, CR and UGI procedures. The result of this participation percentage was due to rearrangement of personnel to other clinical obligations and was associated with clinical restructuring. In summary, the simulator trainings by self-organization of each resident varied immensely and were performed rather irregularly. The average NASA TLX score was 26.9 ± 16.6 for the first 100 hundred robotic operations with a low level of frustration (4.8 ± 4.6) and satisfactory individual performance (6.7 ± 4.6).

Hence, the results of the first phase of development led to several adjustments: smaller OR teams for a period of twelve months (rotation schedule), classification of three procedural clusters (organ systems) to ensure adequate quantity of operations for the scheduled personnel, simulator training concept with specified exercises and new, more time-efficient structure of digital evaluation and data collection with a new web link.

This workup was the basis of the final RoCS of our surgical department. The implementation of the improved RoCS was completed in May 2021 with twelve participating surgeons.

### Curricular structure of RoCS

**Fig. 1:**
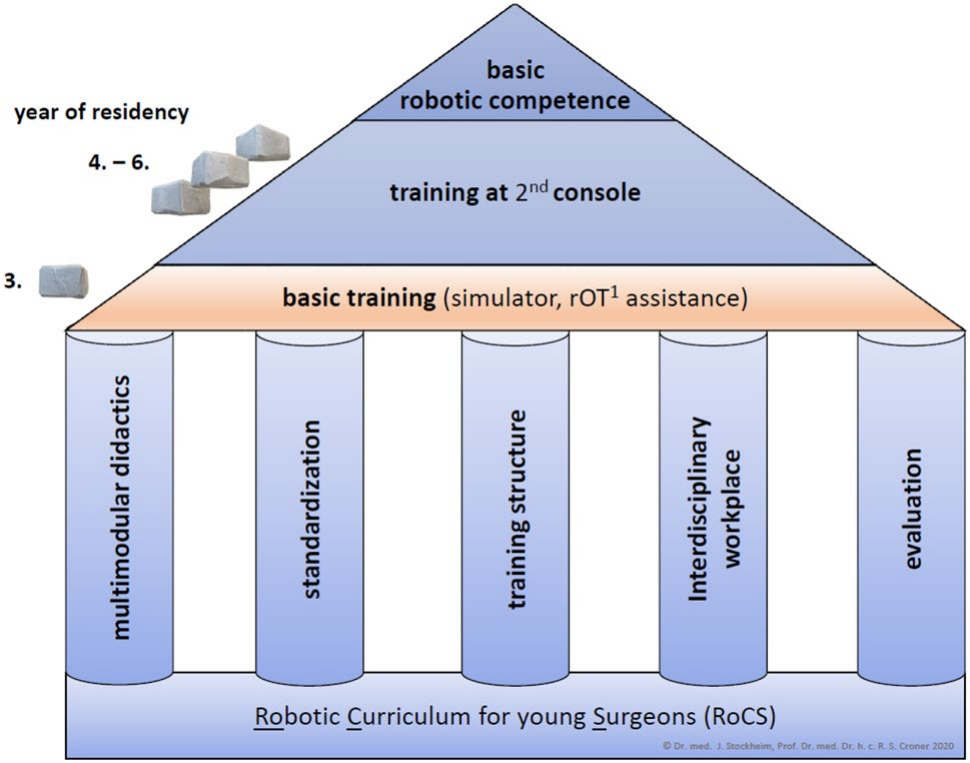
Curricular structure of the “Robotic Curriculum for young Surgeons” (RoCS) ^1^*rOT* robotic operating table

The fundament of the curricular structure of RoCS is based on five modules: multimodal didactics, standardization, training structure, interdisciplinary workplace and evaluation (Fig. 1).

The *didactic module* contains the following elements: 1) know-how (guidelines, digital knowledge, clinic standards, etc.), 2) soft skills (communication, feedback) and 3) surgical technique (practice, simulator exercise, workshops, team training).

The *standardization module* includes perioperative processes, the surgical communication and the specific intraoperative procedural steps. Standardization for the basic training year at the *robotic operating table (rOT)* includes patient positioning, trocar placement, docking, intraoperative assistance and perioperative communication with the console surgeon. According to the *robotic team time-out (rTTO)*, the surgeons discuss the surgical case preoperatively and define intraoperative tasks. This conversation is documented digitally. The surgeons’ orientation for necessary personnel assignment and individual effort depended on the current status in the training concept timeline. By way of continuous feedback collection, the learning process and its outcome can be monitored in surgical and curricular detail. Any discrepancies between the preoperative assignments and the actual surgery are documented.

**Fig. 2:**
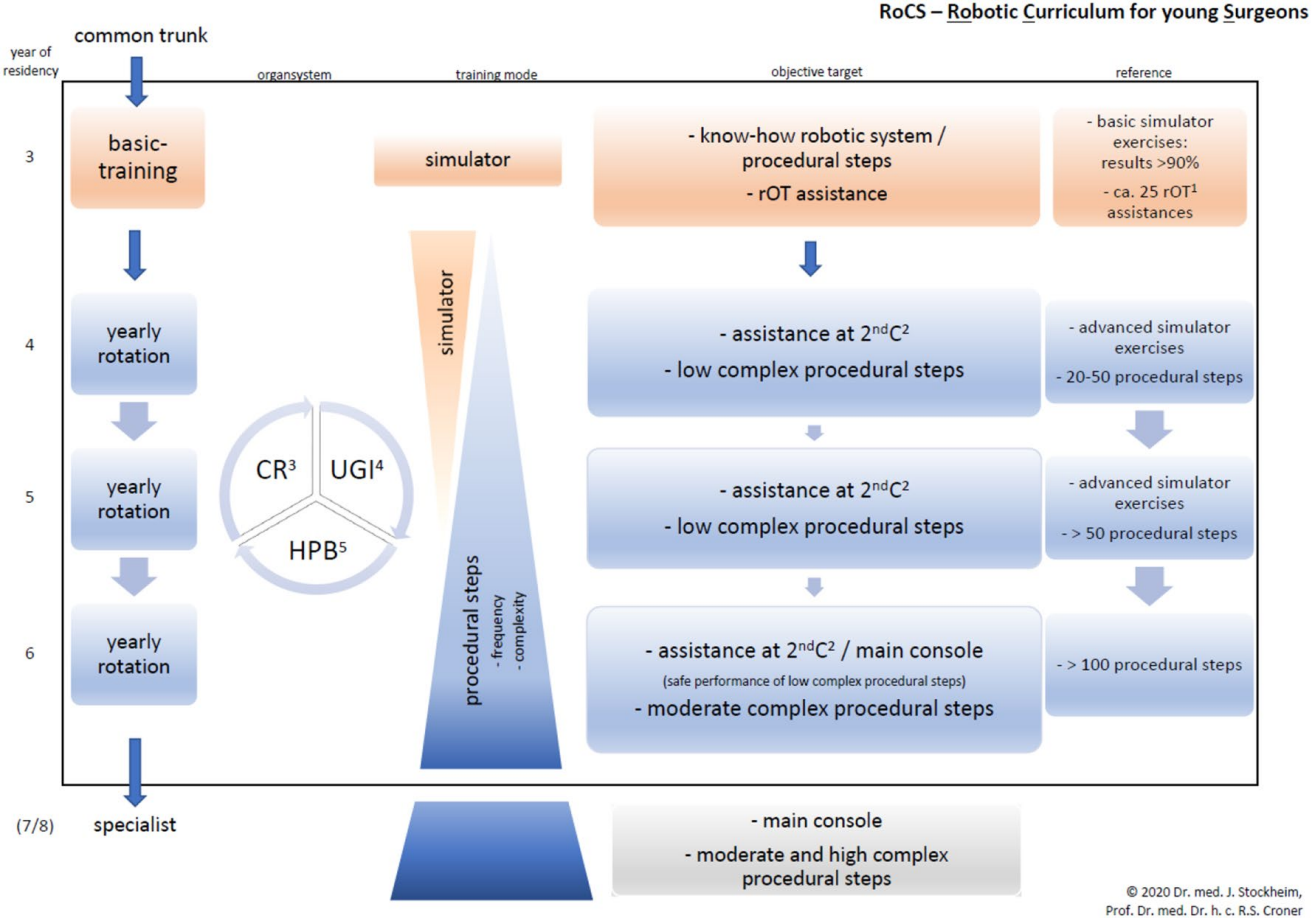
RoCS concept timeline (year of residency, organ system, training mode, objective target, reference ^1^*rOT* robotic operation table; ^2^*2*^*nd*^*C* second console; ^3^*CR* colorectal tract; ^4^*UGI* upper gastrointestinal tract; ^5^*HPB* hepatopancreaticobiliary tract

The main content of the *training structure* module refers to time, content, complexity and flexibility matters. The time-related aspect is represented by a defined timeline for each curricular phase with substantial aspects during the residency (Fig. 2). Content aspects relate to regular rotations (scheduled change of operation site) regarding different visceral organs (*organ systems*). The complexity level of practice and exercises increases with training progress.

The *interdisciplinary workplace* is shaped by the teamwork of the OR staff (surgery, OR nurses, anesthesiologists) as well as the OR setting considering the technical environment, robotic equipment, interdisciplinary workload, hospital policy of efficiency and economy.

Concurrently, the *evaluation* process is assessed digitally using a tablet and local PCs in the operating room (OR).

### Implementation of RoCS: Concept design of robotic training

Aiming basic robotic competence, the training concept design comprises two phases*:* primarily *basic training and assistance at the robotic operating table* and secondary *basic console training*.

Relying on the German system of residency the basic training starts after the “common trunk” and extends one year for surgical rOT assistance. Training of robotic surgical technique is conducted through simulator exercises while practice is assured during intraoperative assistance at the rOT as shown in Fig. 2.

**Fig. 3:**
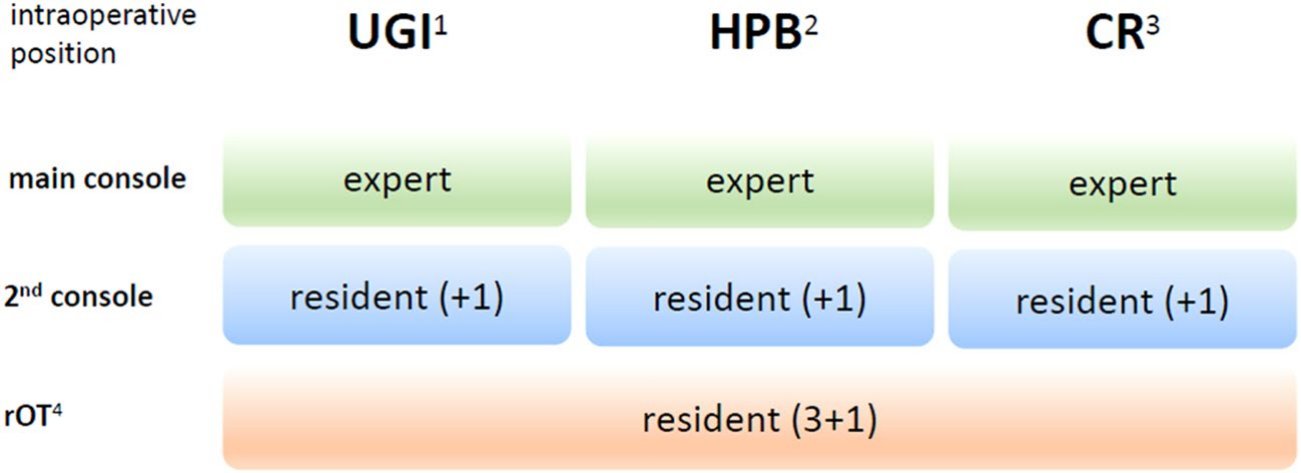
Robotic surgical OR team hierarchy in relation to the organ system and the intraoperative position ^1^*UGI* upper gastrointestinal tract; ^2^*HPB* hepatopancreaticobiliary tract; ^3^*CR* colorectal tract; ^4^*rOT* robotic operation table

Aside from daily assignment of staff, the concept of regular rotation guarantees continuity in the learning process. The *rotation cycles* extend over one year for the assisting residents at the rOT and for the second console, i.e., for a scheduled period of one year, the surgical OR teams are defined. While the assisting surgeon at the second console remains at the position for one particular organ system, the surgeon at the rOT performs assistance for all organ systems during the one year of basic training relying on the clinical organization. The count of reference is around 25 assistances in one year for the rOT (Fig. 3) due to a case load of approximate 100 robotic visceral procedures each year with one operation robot.

According to rotation from the bedside assistance to the assistance at the second robotic console, three assigned surgeons could reach this aim. After the first six months, personnel scheduling for the assistance at the operation table was realized in 82.7% and at the second robotic console in 67.3% of the 52 performed robotic procedures. In general, for each robotic procedure, there was at least one person who was assigned to the RoCS personnel schedule.

The following scheduling structure turned out to be most feasible: clustering of procedures into three organ systems: upper gastrointestinal tract (UGI), liver/pancreas (HPB), colorectal (CR) and appointing three surgeons to each operation (1×main console, 1×assisting surgeon at second console, 1×surgeon at the robotic operating table) (Fig. 3). After finishing their basic training, the novice surgeons advanced to the level of intraoperative assistance at the second console and were trained directly by the expert sitting at the main console. UGI procedures include thoraco-abdominal esophagectomy hiatus hernia repair, (re-)fundoplication and bariatric surgery. Major and minor liver resections (hemi-hepatectomy, split-liver operation, typical and atypical liver resection), left pancreatectomy, pancreatic tail resection, pancreaticoduodenectomy and pancreatic tumor enucleation can be summarized as HPB cluster. The colorectal cluster covers right and left hemicolectomies, sigmoid colon and rectum resections.

During the time of assisting the surgeon at the rOT, a defined set of simulator exercises have to be completed by the novice surgeon with a success rate of at least 90% for each exercise. Therefore, this set of simulator exercises defines the qualification criteria to be upgraded to assistance at the second console assuring the patients safety by basic handling competence. The conceptual center of console training consists of segmentation of each specific procedure in single steps (*procedural steps*) and classification of each step as low, moderate or high complex (*procedural step complexity*). Therefore, young surgeons start to perform low complex (simple) procedural steps initially and independently from the overall complexity of the operation. Over time and with growing experience, the takeover of procedural steps increases in frequency and complexity (Fig. 3). Later on, procedural steps with higher complexity such as moderate complex can be addressed by improved technical skills. To complete the full amount of practice during the initial phase at the second console, advanced simulator exercises have to be performed additionally.

### Evaluation process and feedback loop during RoCS

The *electronic robotic assessment questionnaire (RAQ)* was developed by the curricular team. It included all relevant parameters to ensure analysis of the research interest underlying RoCS. Data collection of patient and operation-related factors were performed in form of a questionnaire pre- and postoperatively. In addition, curricular aspects were implemented for purposes of proving a learning trend. This included the documentation of rTTO, specific intraoperative procedural steps and photo-/ video documentation. Perioperative feedback from the expert surgeon to the assistant and vice versa were included as well. The surgical expertise of each OR team member is evaluated initially during a follow-up every year by a generated questionnaire (Table.2).

For research purposes, the *comprehensive RAQ (cRAQ)* was developed. It combined all necessary information needed for comparison to robotic cases before initiation of RoCS.

## Discussion

First clinical experiences following the initial phase of twelve months led to major improvement of the curricular structures. The process of developing a curricular structure of robotic training for surgical residents without prior experience in robotic surgery is a challenging task [8]. Local resources and limitations need to be addressed individually due to changing financial, technical and personnel factors and situations. It is important to allow full transparency and flexibility within the process [44]. Following principle rules of curriculum design and change management comprehensive projects like RoCS can be implemented successfully without unsafe levels of workload. Methodologically, the idea of our concept was transferred to daily routine by creating a vision supported by a capable guiding team which is recommended [45]. Immediate reward was received by the involved residents due to participating in robotic procedures. Through open communication of the vision of the robotic training during residency, we accomplished motivational persistence and rotation-related scheduling of personnel empowerment of the entire surgical team to follow this vision [45].

On the other hand, this procedure requires repetitive, analytic approach based on ongoing evaluation. Therefore, the validity of the presented work is still limited until further data outcome is given. Malfunction of the documentation system and time delay of the daily adjustments due to rearrangement of surgical staff planning were accepted in the initiation phase of new organizational structures. The effort to implement a regular robotic simulator training interval (e.g., one hour weekly) by self-organization of each resident was not successful. Respecting necessary clinical structures for clinical routine and daily workload, it seems more feasible to mandate and complete a predefined simulator exercises set.

In summary, the principles of simulator, bedside and console training were conceptualized and stands in line with most of the previously published setups [12, 14, 31, 35, 36, 40]. Additionally, the training designs with two curricular phases as shown by Moit et al. [33] and Krause et al. [12] were integrated in a slightly modified way. Hence, the progression of training is based on the stage of surgical residency. Knab et al. offer a curricular step-related evaluation method, but only for fellows and a specific field of visceral surgery [42]. In contrast to RoCS, the available literature does not cover perioperative comprehensive standardized communication and evaluation method addressing all curricular training and evaluation aspects.

In conclusion, we present a novel method which includes all recommended features of a robotic training curriculum which provides a feasible structure for daily routine. The crucial factors are the allocation of robotic teams and clustering procedures in three organ systems (UGI, HPB, and CR). Procedure clustering results in efficient groups to gain practice experience per person. Only Juza et al. [40] described the console training with reference to procedural steps of minimal-invasive cholecystectomy based on perceived level of difficulty. However, RoCS transferred this to every usually performed robotic procedure of UGI, HPB and CR based on current guidelines. Considering this, initial time investment and standardization processes are indispensably for sustainable training effects. Consecutively, the advantage of RoCS is its flexibility and adaptability to internal organization processes of a surgical department due to its independency of OR capacities. The implementation of RoCS caused no interference with the maintenance of surgical case load. Furthermore, it is adaptable to individual careers considering organizational challenges like work–life balance and working schedule models. It marks contrast to voluntary training options during extra hours [12] and represents a target-oriented use of residents’ resources. By definition, gaining robotic surgical competence provides nowadays a career advantage for the next generation of surgeons. We firmly believe that a controlled, systematic training for basic robotic skills is the cornerstone of specialization and can lead to masterclass skills as far as advanced robotic surgery is concerned. Furthermore, it can be used in a multicenter fashion and for different levels of hospital care. In the long turn, RoCS is a flexible, adjustable instrument and can be further improved.

## Conclusion and perspective

The RoCS concept is the first trial to evaluate the specific needs of novice surgeons regarding robotic training. It is a strong tool to standardize procedures and initiate and evaluate interdisciplinary team building. Learning curves including results of rOT assistance, satisfaction of young residents in combination of a workload analysis need to be analyzed. Further studies and clinical cooperation are needed to validate the concept design. So far, we reached the process step of curriculum maintenance and enhancement [8]. It can be considered as the second milestone of this project. The comparison of different robotic systems should be considered as urgent to meet the ongoing personnel, economic, health and technical challenges.
